# Osteocalcin Induces Release of Glucagon-Like Peptide-1 and Thereby Stimulates Insulin Secretion in Mice

**DOI:** 10.1371/journal.pone.0057375

**Published:** 2013-02-20

**Authors:** Akiko Mizokami, Yu Yasutake, Jing Gao, Miho Matsuda, Ichiro Takahashi, Hiroshi Takeuchi, Masato Hirata

**Affiliations:** 1 Laboratory of Molecular and Cellular Biochemistry, Faculty of Dental Science, Kyushu University, Fukuoka, Japan; 2 Division of Orthodontics, Faculty of Dental Science, Kyushu University, Fukuoka, Japan; 3 Division of Applied Pharmacology, Kyushu Dental College, Kitakyushu, Japan; University of Ulster, United Kingdom

## Abstract

The uncarboxylated form (ucOC), but not the γ-carboxylated form (GlaOC), of the bone-derived protein osteocalcin stimulates insulin secretion and regulates energy metabolism in insulin target tissues. Glucagon-like peptide–1 (GLP-1) is an insulin secretagogue that is released from the gut in response to food intake. We have now found that Gprc6a, a putative ucOC receptor, is expressed in epithelial cells of the mouse small intestine as well as in STC-1 enteroendocrine cells. Secretion of GLP-1 by STC-1 cells was stimulated by ucOC but not by GlaOC. The serum GLP-1 concentration in mice was increased by intraperitoneal or oral administration of ucOC, whereas GlaOC was effective in this regard only after oral application. Serum insulin levels were also increased by ucOC, and this effect was potentiated by an inhibitor of dipeptidyl peptidase IV and blocked by a GLP-1 receptor antagonist. Intravenous injection of ucOC in mice increased the serum GLP-1 concentration, and also increased the serum level of insulin. Our results suggest that ucOC acts *via* Gprc6a to induce GLP-1 release from the gut, and that the stimulatory effect of ucOC on insulin secretion is largely mediated by GLP-1.

## Introduction

Bone is one of the largest organs in the human body and undergoes remodeling both during childhood and throughout adulthood. Bone remodeling, characterized by repetitive bone resorption by osteoclasts and bone formation by osteoblasts, is tightly regulated at the local level by cytokines produced by bone cells as well as at the systemic level by hormones and neuropeptides [1], [2]. Bone is also under the influence of hormones that regulate energy metabolism such as leptin, an adipocyte-derived hormone that regulates appetite and energy expenditure and which modulates postnatal bone acquisition through activation of several signaling pathways [3], [4]. Another such hormone is insulin. Osteoblasts thus express functional insulin receptors, the stimulation of which in primary osteoblasts or osteoblast-like cell lines results in the up-regulation of bone anabolic markers including collagen synthesis, alkaline phosphatase production, and glucose uptake [5]–[7].

Bone is not merely a passive tissue that is subject to external influences, however. It is also an active endocrine organ that produces at least two hormones, fibroblast growth factor 23 [8] and osteocalcin [9]. Osteocalcin (OC) increases insulin production and sensitivity and thereby promotes glucose utilization and energy expenditure [9]. It also undergoes vitamin K–dependent carboxylation on three glutamic acid residues, which gives rise to GlaOC and confers a high affinity for the bone matrix. A small proportion of OC molecules remain uncarboxylated and are secreted into the circulation [10]. The acidic pH of the bone-resorbing niche promotes the decarboxylation of GlaOC [11], and the resulting uncarboxylated osteocalcin (ucOC) is responsible for the stimulation of insulin secretion. In turn, insulin signaling in osteoblasts promotes bone formation by suppressing the expression of Twist2, an inhibitor of osteoblast development, and increases the expression of OC [12]. Furthermore, insulin signaling down-regulates the expression of osteoprotegerin, an osteoblast-specific inhibitor of RANKL (receptor activator of nuclear factor-κB ligand), and thereby promotes bone resorption by osteoclasts, resulting in the release of active ucOC [11] and completing a feed-forward loop.

Incretin hormones also promote the secretion of insulin from pancreatic β cells in a glucose-dependent manner [13]. Glucagon-like peptide–1 (GLP-1), one of the incretin hormones, is produced by enteroendocrine L cells of the small intestine and is secreted into the circulation in response to nutrient ingestion [14]. GLP-1 achieves its insulinotropic effect by binding to its specific receptor and thereby increasing the cytosolic concentrations of cAMP and Ca^2+^ in β cells [15]. It also stimulates β cell proliferation as well as protects the cells from apoptosis [13]. In the present study, we have therefore investigated whether, in addition to its direct effect on the pancreas, OC might increase GLP-1 secretion.

## Materials and Methods

### Cell culture

STC-1 cells (kindly provided from Dr. G. Tsujimoto, Kyoto University) [16], a mouse enteroendocrine cell line were cultured under a humidified atmosphere of 5% CO_2_ at 37 °C in Dulbecco's modified Eagle's medium supplemented with 15% horse serum, 5% fetal bovine serum, penicillin (100 U/ml), and streptomycin (0.1 mg/ml). The cells were routinely passaged at 80 to 90% confluence.

### RT-PCR analysis

Total RNA (2 µg) isolated with an RNeasy Mini Kit (Qiagen, Valencia, CA) was subjected to RT-PCR (reverse-transcriptase polymerase chain reaction) analysis with the use of a ReverTra Ace kit (Toyobo, Osaka, Japan) and with the Gprc6a primers 5′-CCAGACGACCACAAATCCAG-3′ (forward) and 5′-GATTCATAACTCACCTGT-3′ (reverse).

### Immunohistochemistry

Mouse small intestine was dissected to the upper, middle, and lower thirds (as duodenum, jejunum, and ileum, respectively), fixed in 4% paraformaldehyde, dehydrated with a series of ethanol solutions, embedded in paraffin, and sectioned at a thickness of 6 µm. The sections were then depleted of paraffin and rehydrated with phosphate-buffered saline. Antigen retrieval was performed with an autoclave for 5 min at 121 °C in 10 mM sodium citrate buffer (pH 6.0), and nonspecific protein binding was blocked by incubation of the sections with 10% goat serum in phosphate-buffered saline. The sections were then incubated overnight at 4 °C with rabbit antibodies to Gprc6a (1∶500 dilution, PAB16273; Abnova, Taipei, Taiwan) or with rabbit γ-globulin (1∶500 dilution; Jackson ImmunoResearch Laboratories, West Grove, PA) as a negative control. Immune complexes were detected with Alexa Fluor 488-labeled goat antibodies to rabbit immunoglobulin G (1∶1000 dilution; Molecular Probes, Carlsbad, CA), and nuclei were stained with Hoechst 33342 (Dojindo Laboratory, Kumamoto, Japan). Sections of mouse ileum were incubated overnight at 4 °C with rabbit antibodies to Gprc6a (or with control γ-glubulin), after which immune complexes were detected with biotinylated goat antibodies to rabbit IgG (1∶1000 dilution; Vector Laboratories, Burlingame, CA) and a Vector ABC Elite kit plus ImmPact DAB (Vector Laboratories). The sections were counterstained with Mayer's hematoxylin (Muto Pure Chemicals, Tokyo, Japan) and examined with a BZ-9000 microscope (Keyence, Osaka, Japan).

### Preparation of recombinant ucOC

An expression vector for a glutathione *S*-transferase-ucOC fusion protein was constructed by introduction of mouse OC cDNA into the vector pGEX-4T-1 (GE Healthcare, Buckinghamshire, UK). The fusion protein was produced in bacteria and purified with glutathione-Sepharose beads (GE Healthcare) according to standard procedures. The final washes of the beads were performed with a buffer containing 0.1% Triton X-114 (Sigma-Aldrich, St. Louis, MO) and then with the same buffer without detergent in order to remove endotoxin [17]. The ucOC moiety was then cleaved from glutathione *S*-transferase with thrombin, which was then removed from the preparation with the use of a benzamidine Sepharose 4 Fast Flow column (GE Healthcare). The purity of the ucOC preparation was assessed by sodium dodecyl sulfate-polyacrylamide gel electrophoresis with Tris-Tricine and staining with Coomassie blue, and the concentration and integrity of the protein were determined with an enzyme-linked immunosorbent assay (ELISA) kit for mouse OC (Biomedical Technologies, Stoughton, MA). For preparation of ^125^I-labeled ucOC, glutathione *S*-transferase-ucOC immobilized on glutathione-Sepharose was exposed to Na^125^I with the use of an iodination tube (Thermo Scientific, Rockford, IL). The beads were washed and exposed to thrombin, and the released ^125^I-labeled ucOC was separated from free Na^125^I with a PD10 MidiTrap G-10 (GE Healthcare).

### GLP-1 secretion from STC-1 cells

STC-1 cells in a six-well plate were washed three times and then incubated with recombinant mouse ucOC or with GlaOC (ANA Spec, Fremont, CA) in Hanks' balanced salt solution for 60 min at 37 °C. The culture supernatants were then assayed for GLP-1 with ELISA kit (Shibayagi, Gunma, Japan).

### Animal experiments

Male C57BL/6J mice (Jackson Laboratory, Bar Harbor, ME) were maintained in a specific pathogen-free facility under a 12 h-light, 12 h-dark cycle and were moved to a conventional facility for experiments at 6 to 8 weeks of age. All animal experiments were approved by the animal ethics committee of Kyushu University. Animals were anesthetized with sevoflurane and blood (maximally up to 100 µl) was collected from the orbital plexus of mice treated as indicated. Dipeptidyl peptidase (DPP)-IV inhibitor (Merck-Millipore, Billerica, MA) was added to the samples to a final concentration of 100 µM. Serum prepared from the blood samples was assayed for GLP-1 or insulin with ELISA kits obtained from Shibayagi and Mercodia (Uppsala, Sweden), respectively. Exendin-4 and exendin(9–39) used in animal experiments were obtained from Sigma-Aldrich and Bachem AG, Bubendorf, Switzerland, respectively.

### Measurement of serum OC concentrations

Serum OC concentrations were measured with ELISA kit for mouse OC (Biomedical Technologies), which is formulated for both ucOC and GlaOC, with the two forms being discriminated with the use of hydroxyapatite. Serum (50 µl) was thus mixed with 10 mg of hydroxyapatite (Sigma-Aldrich) and rotated at 4 °C for 1 h, after which the hydroxyapatite (and bound GlaOC) was removed by centrifugation and the supernatant was assayed for ucOC.

### Statistical analysis

Data are presented as means ± SEM and were analyzed as indicated. A *P* value of <0.05 was considered statistically significant.

## Results

### Gprc6a expression in intestinal cells

**Figure 1:**
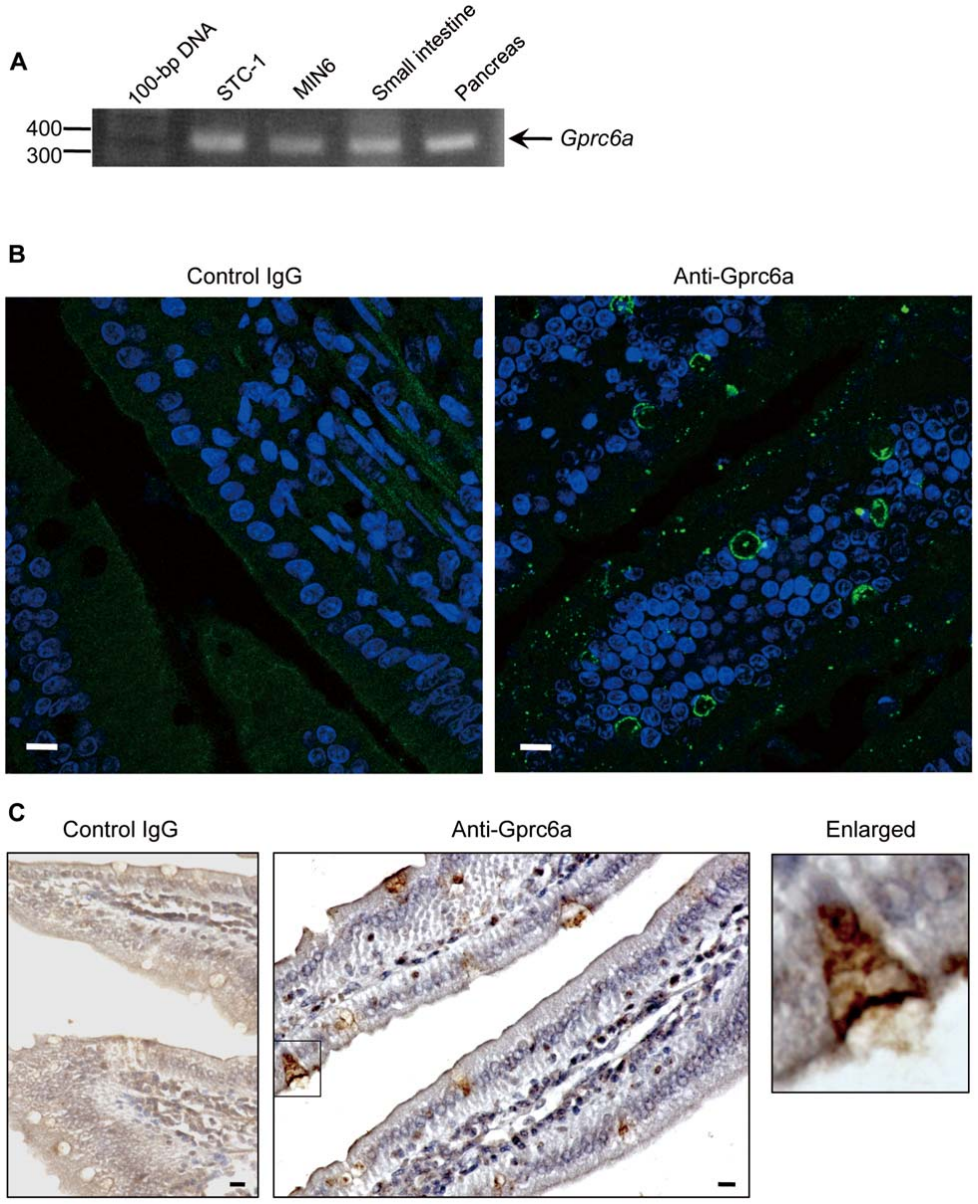
Expression of Gprc6a in mouse small intestine. (**A**) Semiquantitative RT-PCR analysis of Gprc6a mRNA in STC-1 and MIN6 cells as well as in mouse small intestine and pancreas. (**B**) Immunofluorescence analysis of Gprc6a expression (green) in mouse ileum. Rabbit γ-globulin was used as a negative control, and nuclei were stained with Hoechst 33342 (blue). (**C**) Gprc6a immunostaining by ABC techniques (brown), counterstained with hematoxylin. A higher magnificent image of cell indicated by a rectangle is shown in the right. Bars, 10 µm.

We first examined whether the putative OC receptor Gprc6a [18], [19] is expressed in intestinal cells. RT-PCR analysis revealed the presence of Gprc6a mRNA in STC-1 cells and mouse small intestine as well as in mouse pancreas and the mouse β cell line MIN6 examined as positive controls [18] (Fig. 1A). The identity of the observed PCR product was confirmed by sequencing. Immunostaining for Gprc6a in the mouse ileum showed that the immunoreactivities were found in the epithelial cells in a dotted fashion as well as around goblet cells (Fig. 1B). Immunohistochemical staining further revealed that Gprc6a was detected in epithelial membranes of the small intestine, and the immunoreactivities were found in both apical and basolateral membranes in some epithelial cells (Fig. 1C and the enlargement).

### Effect of OC on GLP-1 secretion by STC-1 cells

**Figure 2:**
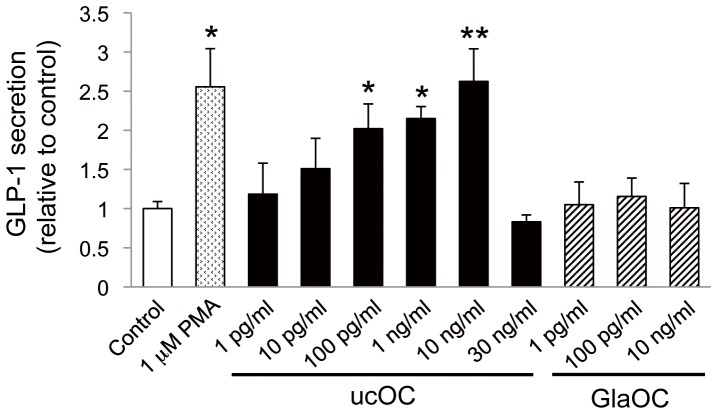
OC-induced GLP-1 secretion in STC-1 cells. STC-1 cells were incubated for 60 min with vehicle (control) or the indicated concentrations of ucOC, GlaOC, or PMA. Data are expressed relative to the control value (33.1 pg per 1 × 10^5^ cells) and are means ± SEM from four to eight independent experiments. *P < 0.05, **P < 0.01 *versus* control (Student's *t* test).

We then examined whether OC might affect GLP-1 secretion by STC-1 cells. Recombinant ucOC induced GLP-1 secretion in a concentration-dependent manner, with this effect being significant at 100 pg/ml to 10 ng/ml (∼20 pM to ∼2 nM) but no longer apparent at 30 ng/ml for unknown reason(s) (but see Discussion) (Fig. 2). The maximal effect of ucOC was similar to the effect of 1 µM phorbol 12-myristate 13-acetate (PMA) examined as a positive control [16]. In contrast, GlaOC did not affect GLP-1 secretion at the concentrations examined.

### OC-induced GLP-1 secretion *in vivo*

**Figure 3:**
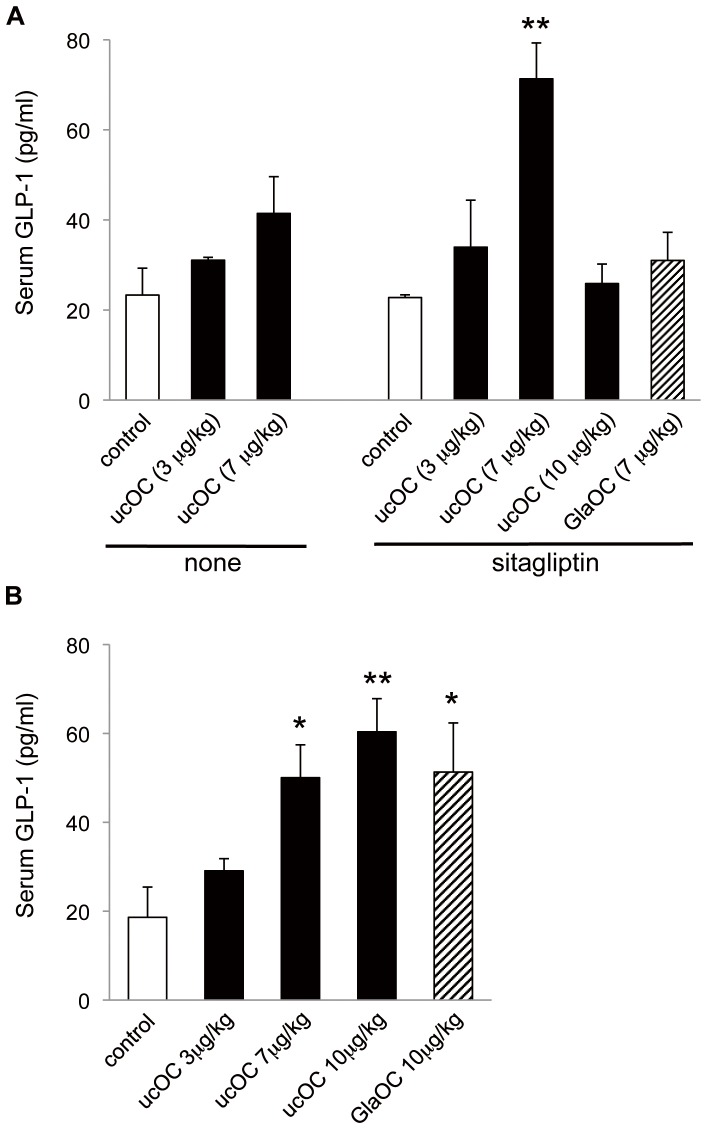
Effect of intraperitoneal or oral administration of OC on serum GLP-1 concentration. (**A**) Mice were deprived of food for 5 h and then injected intraperitoneally with saline (control) or the indicated doses of recombinant ucOC, of GlaOC. Blood was collected 20 min later for determination of the serum concentration of GLP-1. Part of mice was orally administered with sitagliptin (1 µg/kg) 30 min before injection of OC. (**B**) Mice deprived of food for 5 h were treated orally with saline (control) or the indicated doses of ucOC or GlaOC. Blood samples collected 20 min later were assayed for serum GLP-1 concentration. All data are means ± SEM for five to seven mice. *P < 0.05, **P < 0.01 *versus* control (Student's *t* test).

The effect of ucOC on GLP-1 secretion *in vivo* was examined 20 min after the 5 h-fast with male C57BL/6J mice. Intraperitoneal administration of ucOC induced an increase in the serum concentration of GLP-1, but with only statistically insignificance (Fig. 3A). Given that GLP-1 has a short half-life *in vivo* as a result of its degradation by DPP-IV [20], we examined the effect of ucOC on the serum GLP-1 level in mice first treated orally with the DPP-IV inhibitor sitagliptin. Intraperitoneal administration of ucOC at 7 µg/kg in such mice induced a significant increase in the serum GLP-1 concentration (Fig. 3A). However, injection of ucOC at 10 µg/kg had no such effect, reminiscent of the concentration dependence of the stimulation of GLP-1 secretion by ucOC in STC-1 cells (Fig. 2). We also found that intraperitoneal administration of GlaOC did not increase the serum GLP-1 level in sitagliptin-pretreated mice (Fig. 3A).

We then examined the effect of oral administration of OC on circulating GLP-1 levels. Such administration of ucOC increased the serum concentration of GLP-1 in a dose-dependent manner, even in the absence of sitagliptin pretreatment, and a similar effect was apparent after oral administration of GlaOC (Fig. 3B) in a clear contrast to that by intraperitoneal administration.

### OC-induced insulin secretion *in vivo* and the effect of blockade of GLP-1 action

**Figure 4:**
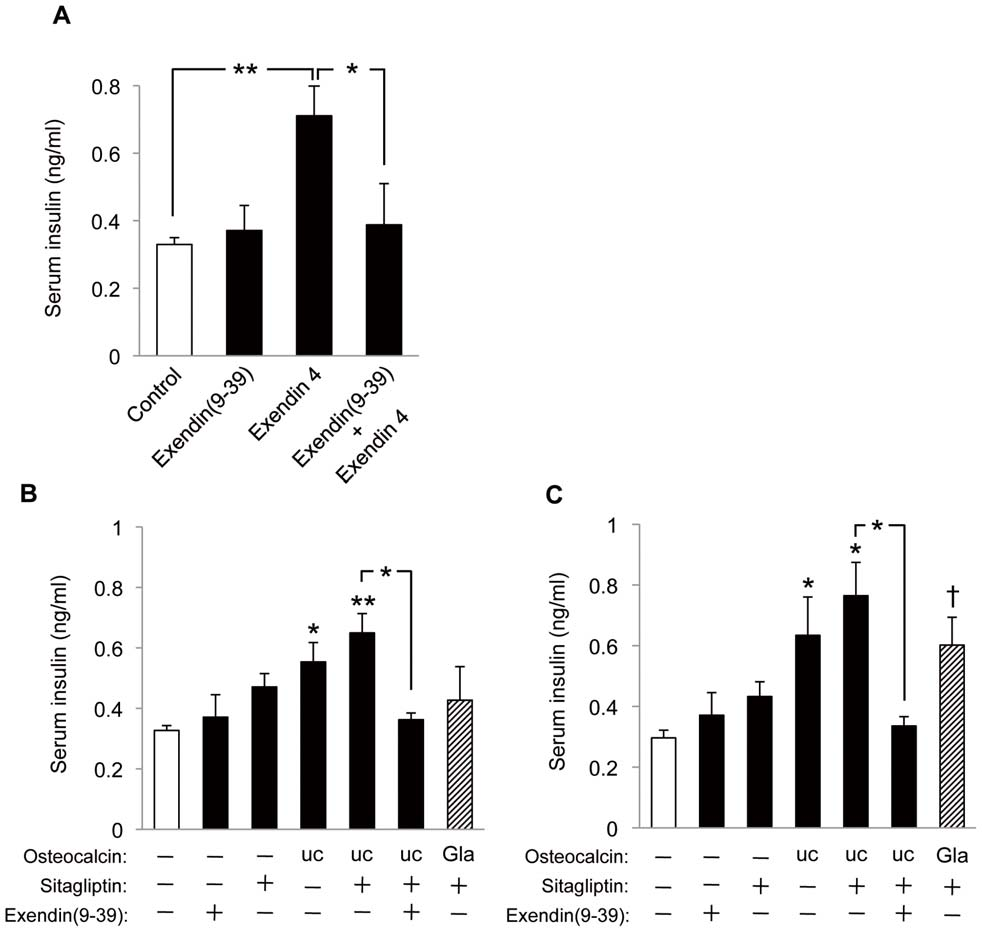
Effects of the GLP-1 receptor antagonist exendin(9–39) on insulin secretion induced by OC. (**A**) Mice deprived of food for 5 h (blood glucose level, 103.5 mg/dl) were injected intraperitoneally with saline or exendin-4 (25 nmol/kg) at 30 min after intraperitoneal injection with saline or exendin(9–39) (25 nmol/kg). Blood samples were collected 20 min after exendin-4 injection for determination of the serum insulin concentration. (**B**, **C**) Mice deprived of food for 5 h (blood glucose level, 103.5 mg/dl) were administered sitagliptin (1 µg/kg) or saline orally and then 30 min later were treated with ucOC or GlaOC intraperitoneally (7 µg/kg) (**B**) or orally (10 µg/kg) (**C**) in combination with intraperitoneal injection of exendin(9–39) (25 nmol/kg) or saline. Blood samples were collected 20 min after OC administration for assay of serum insulin. All data are means ± SEM for four to six mice. *P < 0.05, **P < 0.01, †*P*  =  0.06 *versus* control (open bar) or for the indicated comparisons (Student's *t* test).

Serum insulin levels were previously shown to be increased by ucOC in a manner thought to be dependent on a direct effect on pancreatic β cells [9], [21]. To evaluate whether the effect of ucOC on GLP-1 secretion observed in the present study might contribute to the insulinotropic action of ucOC, we examined the effect of exendin(9–39), a GLP-1 receptor antagonist [22]. We first confirmed that exendin(9–39) acted as a GLP-1 receptor antagonist in our experiments. Exendin-4 binds to and activates GLP-1 receptors [22], [23], and we found that intraperitoneal administration of exendin-4 increased the serum insulin concentration in mice in a manner sensitive to prior injection of exendin(9–39) at a dose (25 nmol/kg) previously shown [24] to fully antagonize GLP-1 signaling in mice (Fig. 4A).

We then treated mice intraperitoneally or orally with OC in the presence or absence of exendin(9–39) as well as with or without prior oral administration of sitagliptin (Fig. 4B, C). Sitagliptin itself induced a small increase in the serum insulin concentration, which was likely attributable to basal secretion of GLP-1 given that it was blocked by administration of exendin(9–39) (data not shown). Intraperitoneal injection of ucOC (7 µg/kg) alone induced a significant increase in the serum insulin concentration that was enhanced by sitagliptin, and this combined effect of both agents was abolished by exendin(9–39) (Fig. 4B). Intraperitoneal administration of GlaOC had no effect on serum insulin, similar to its lack of effect on serum GLP-1. Essentially identical results were observed with oral administration of ucOC (10 µg/kg), whereas oral GlaOC tended to increase serum insulin, although this effect did not achieve statistical significance (Fig. 4C). In addition, oral administration of ucOC appeared to be more effective in this regard than the intarperitoneal administration, reminiscent of the effect of oral ucOC in serum GLP-1 levels (Fig. 3). Together, these results thus indicated that OC increases the serum concentration of insulin in a manner almost completely dependent on GLP-1.

### Serum concentration of OC

**Figure 5:**
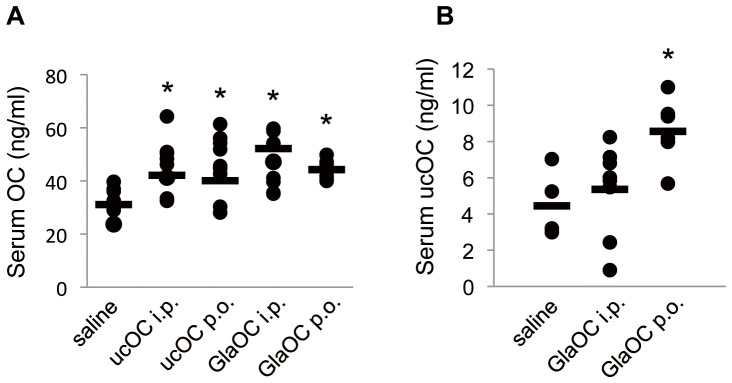
Serum levels of total OC and ucOC after intraperitoneal or oral administration of ucOC or GlaOC. Serum levels of total OC (**A**) or ucOC (**B**) were measured 20 min after administration of ucOC or GlaOC at a dose of 7 µg/kg intraperitoneally (i.p.) or 10 µg/kg orally (p.o.) in mice that had been deprived of food for 5 h; black circles indicate values for individual mice, with the horizontal bars representing the mean values. *P < 0.05 versus saline-treated animals [one-way ANOVA followed by either Dunnett's multiple-comparison test (**A**) or Tukey's HSD post hoc test (**B**)].

We next measured the circulating levels of total OC (both Gla- and uc-forms) and of ucOC after intraperitoneal or oral administration of either uc- or Gla-form of OC. We found that the total OC concentration in serum was increased to similar extents after administration of ucOC or GlaOC either intraperitoneally or orally (Fig. 5A), suggesting that orally administered OC was absorbed from the alimentary canal in its intact form and entered the bloodstream, and that such absorbed ucOC might therefore trigger the secretion of GLP-1. We also found that the serum level of ucOC was increased significantly after oral administration of GlaOC but not after its intraperitoneal administration (Fig. 5B). The total OC concentration in serum increased from ∼31 to ∼42 ng/ml after oral application of ucOC or GlaOC at 10 µg/kg, indicating that ∼4% of administered OC was absorbed into the circulation, assuming a mean blood volume of 2 ml (hematocrit of ∼50%) and mean body weight of 25.4 g.

### Absorption of ucOC

**Figure 6:**
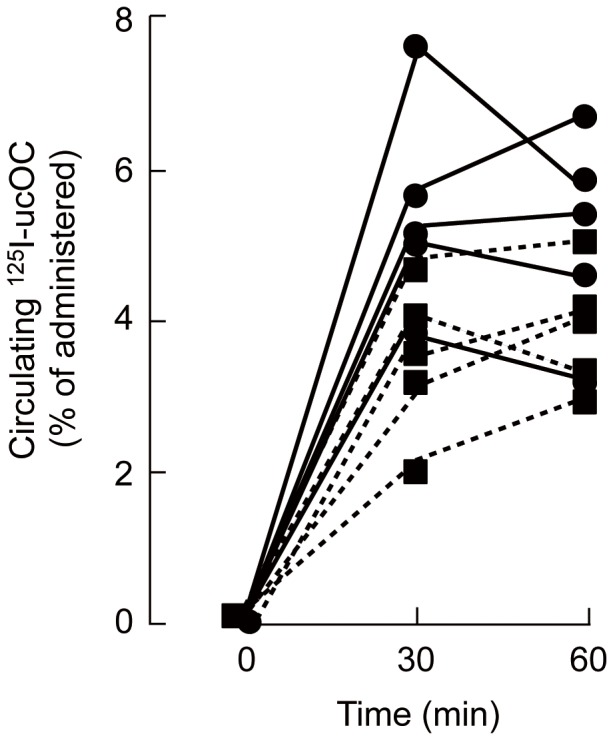
Absorption of ^125^I-labeled ucOC into the systemic circulation. Mice were administered ^125^I-labeled ucOC (5 µg/kg, 4,000 to 6,000 cpm) intraperitoneally (circles, n  =  5) or orally (squares, n  =  5), and blood was collected into tubes containing 5 mM EDTA (to prevent coagulation) at 30 and 60 min thereafter for measurement of radioactivity. The percentage of the administered radioactivity present in the circulation was calculated assuming a total blood volume of 2 ml. The amount of radioactivity per wet tissue weight for the thymus of the treated mice was found to be similar to that for other organs such as the liver, whereas the thymus of mice administered Na^125^I instead of ^125^I-ucOC preferentially accumulated radioactivity. These findings indicate that the radioactivity in the ucOC preparation was present mostly in the form of the labeled peptide rather than as free Na^125^I.

To examine the direct absorption of ucOC from the intestine into the circulation, discriminating it from endogenous OC potentially released by an unknown mechanism in response to OC administration, we examined whether ^125^I-labeled ucOC applied intraperitoneally or orally enters the systemic circulation (Fig. 6). Mice were thus administered ^125^I-labeled ucOC intraperitoneally or orally, and blood was collected at 30 and 60 min thereafter for measurement of radioactivity. Radioactivity indicative of the presence of ^125^I-ucOC in blood was detected after either intraperitoneal or oral application. Gross absorption ranged from ∼2 to 8% of the administered radioactivity at 30 min, with the values for intraperitoneal injection tending to be slightly higher than those for oral administration. The values had increased further or decreased to a small extent at 60 min.

### Effects of intravenous administration of ucOC on serum GLP-1 and insulin levels

**Figure 7:**
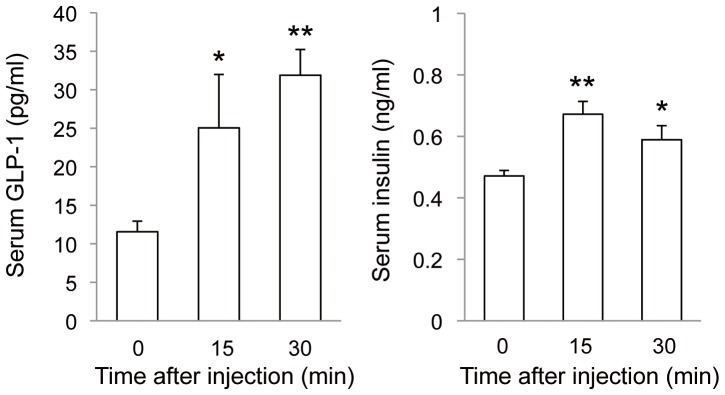
Effects of intravenous injection of ucOC on serum GLP-1 and insulin levels. Mice deprived of food for 5 h (blood glucose level, 103.5 mg/dl) were injected with ucOC (30 ng per animal) *via* the tail vein, and blood samples were collected 15 and 30 min thereafter for measurement of the serum concentrations of GLP-1 and insulin. Data are means ± SEM for four to six mice. *P < 0.05, **P < 0.01 *versus* time 0 (Student's *t* test).

Our results thus indicated that orally administered OC is absorbed into the systemic circulation and thereby triggers secretion of GLP-1. We finally analyzed the effects of intravenous injection of 30 ng of ucOC on the serum levels of GLP-1 and insulin. This dose would be expected to result in a serum concentration of ∼30 ng/ml at the initial, about three times the increase in the serum OC concentration (∼10 ng/ml) induced by intraperitoneal or oral application of OC at 7 or 10 µg/kg, respectively (Fig. 5A). For mice whose blood glucose level was 103.5 ± 3.5 mg/dl even after the deprivation of food for 5 h, the serum concentration of GLP-1 was significantly increased at 15 min and further increased at 30 min after ucOC injection, and that of insulin increased as well (Fig. 7). Injection of GlaOC into the tail vein had no effect on serum GLP-1 or insulin levels in fasted mice (data not shown). Intravenous injection of ucOC at a high dose of 300 ng also had no effect on the serum insulin concentration in fasted mice (data not shown). These data suggested that circulating ucOC at an appropriate concentration triggers the secretion of GLP-1, which results in the secretion of insulin.

## Discussion

Intraperitoneal injection of recombinant ucOC was previously shown to increase the serum insulin level [18], [25], with this effect having been thought to reflect a direct action on the pancreas [9], [21]. We here observed a similar increase in the serum insulin concentration after the administration of ucOC, but this effect was augmented in the presence of the DPP-IV inhibitor sitagliptin and abolished by the GLP-1 receptor antagonist exendin(9–39). These results thus indicate that the increase in the serum concentration of insulin induced by ucOC is mediated largely by GLP-1, and therefore this effect of ucOC might coexist with its direct effect on the pancreas.

We also found that oral application of OC in either the uc- or Gla-form induced the secretion of GLP-1, an effect likely mediated through the putative OC receptor Gprc6a. Gprc6a mRNA was detected in mouse enteroendocrine STC-1 cells and small intestine as well as in the pancreas and MIN6 pancreatic β cells. Gprc6a immunoreactivity was also detected in both apical and basolateral membranes of epithelial cells of the small intestine, suggesting that ucOC might act on the epithelial cells both from within the intestinal lumen as well as from within the microvasculature of intestinal cilia. However, more detailed immunohistochemical studies are apparently needed to exhibit the co-localization with GLP peptides. The uc-form of OC injected intraperitoneally likely enters the circulation through mesenteric veins and thereby consecutively triggers the secretion of GLP-1 and insulin through interaction with Gprc6a in the intestinal epithelial cells. We initially assumed that orally administered OC would be degraded by protein- and peptide-hydrolyzing enzymes present in gastric and enteric juices, and that OC molecules that escaped such degradation or degradation products themselves might stimulate GLP-1 secretion through interaction with Gprc6a present from the alimental canal. However, the serum level of intact OC (not degradation products thereof) was increased after oral administration of OC, raising the possibility that orally administered ucOC also acts to elicit GLP-1 secretion from within the ciliary microvasculature, thus leading a greater effect than intraperitoneal administration. Our results further indicated that OC, a peptide composed of 46 amino acids, is absorbed from the intestinal lumen into the circulation in its original form to only a limited extent of ∼4% when orally administered at 10 µg/kg. Oral application at two- or fivefold higher doses yielded similar serum levels (unpublished results), indicative of absorption rates of 2% and 0.8%, respectively. Direct absorption of ucOC into the systemic circulation was supported by measurement of radioactivity after intraperitoneal or oral application of ^125^I-labeled ucOC. The blood radioactivity might also contain the degraded form of [^125^I]ucOC, but the ratio of absorption was comparable to that measured by ELISA, indicating that the majority of the radioactivity represents the intact ucOC. Insulin as well as other peptides including calcitonin, vasopressin, and thyrotropin-releasing hormone analogs are also absorbed from the intestine into the bloodstream at a rate of ∼5% [26]. The oral bioavailability of peptide and protein drugs is generally poor as a result of their extensive degradation by proteases in the gastrointestinal tract or their limited ability to cross the intestinal mucosa.

The increase in the serum OC level after intraperitoneal or oral administration of ucOC at 7 or 10 µg/kg, respectively, was ∼8.3 ng/ml (from 31.1 to 39.5 or 39.2 ng/ml), which is equivalent to ∼0.75 nM in blood based on a hematocrit of ∼50% and corresponds to the upper end of the concentration range of ucOC found to be effective in stimulation of GLP-1 secretion from STC-1 cells. The increase in the serum concentration of ucOC after oral administration of GlaOC at 10 µg/kg was ∼4.6 ng/ml (from 4.1 to 8.7 ng/ml), which is equivalent to ∼0.4 nM in blood. Intravenous injection of ucOC at 30 ng, yielding an initial concentration of 2.7 nM in blood, triggered the secretion of GLP-1. However, intravenous injection of ucOC at 300 ng, yielding a blood concentration of 28 nM, did not induce insulin secretion. These quantitative analyses indicate that the physiological range of ucOC concentration effective for induction of the secretion of GLP-1 and insulin is achieved in the bloodstream after intraperitoneal or oral application of OC.

ucOC has been recognized as a hormone that promotes β cell proliferation as well as insulin expression and secretion by β cells of the pancreas [9], [21]. It also increases the insulin sensitivity of peripheral tissues including skeletal muscle and adipose tissue, resulting in increased energy expenditure [11], [12]. The therapeutic potential of ucOC for type 2 diabetes was demonstrated in mice subjected to its continuous administration *via* a subcutaneous osmotic pump [21] or to its daily intraperitoneal injection [25]. Intraperitoneal injection is not a preferred route of drug administration, however. We have now shown that oral administration of ucOC induces levels of GLP-1 and insulin secretion similar to those apparent after its intraperitoneal injection. Oral administration of ucOC also has the advantage that the increase in serum OC level was found to be similar for low and high doses of ucOC, probably because of limited absorption, and thus this route avoids the ineffectiveness of high doses of ucOC with regard to the induction of GLP-1 secretion that was apparent after intraperitoneal administration. At concentrations of >10 ng/ml and intraperitoneal doses of >7 µg/kg for STC-1 cells and mice, respectively, the effect of ucOC on GLP-1 secretion was markedly diminished. Exposure of pancreatic β cells or MIN6 cells to ucOC was previously found to increase insulin gene expression at a low concentration but to inhibit it at a high concentration [21]. The reason for these differences in the effects of ucOC at low and high concentrations remains currently unknown, but might be attributed to the reduced expression of cyclin D2 and cdk4 by high dose of OC, which are necessary for β cell proliferation *in vivo* [27], [28]. Cyclin D2 is also expressed in the small intestine, regulating the proliferation of epithelial cells [29].

GlaOC was effective in inducing GLP-1 secretion only when administered orally, suggesting that oral administration of bone extracts or dried bone powder containing GlaOC might be similarly effective. Previous studies [9], [11] as well as our present results show that ucOC is the form of OC actually responsible for inducing the secretion of GLP-1 or insulin, indicating that GlaOC is decarboxylated after its oral administration. Given that acidic conditions promote the decarboxylation of GlaOC *in vitro* [11], [30], stomach acidity might be responsible for this process *in vivo*. The serum level of ucOC was thus increased and the secretion of GLP-1 was induced after oral administration of GlaOC but not after its intraperitoneal administration.

In summary, our results indicate that ucOC acts both from within the lumen of the small intestine and from within the ciliary microvasculature to stimulate GLP-1 secretion through interaction with the G protein–coupled receptor Gprc6a. The resulting increase in the circulating concentration of GLP-1 is then largely responsible for the stimulation of insulin secretion by ucOC. Given that GLP-1 is clinically effective for the treatment of type 2 diabetes, OC itself, or chemicals or foods containing OC as an ingredient, might also prove efficacious for the treatment of this condition.
